# Factors Affecting Health-Related Quality of Life and Physical Activity after Liver Transplantation for Autoimmune and Nonautoimmune Liver Diseases: A Prospective, Single Centre Study

**DOI:** 10.1155/2014/738297

**Published:** 2014-03-04

**Authors:** Katarzyna Kotarska, Ewa Wunsch, Agnieszka Kempińska-Podhorodecka, Joanna Raszeja-Wyszomirska, Dimitrios P. Bogdanos, Maciej Wójcicki, Piotr Milkiewicz

**Affiliations:** ^1^Department of Physical Culture and Health Promotion, University of Szczecin, 70 067 Szczecin, Poland; ^2^Liver Research Laboratories, Pomeranian Medical University, 70 111 Szczecin, Poland; ^3^Medical Biology Laboratory, Pomeranian Medical University, 70 111 Szczecin, Poland; ^4^Liver and Internal Medicine Unit, Department of General, Transplant and Liver Surgery of the Medical University of Warsaw 02 097 Warsaw, Poland; ^5^Institute of Liver Studies, Division of Liver Transplantation and Mucosal Biology, King's College London School of Medicine, Denmark Hill Campus, London SE5 9RS, UK

## Abstract

*Background/Aim.* With the improvement of the outcomes after liver transplantation (LTx), health-related quality of life (HRQoL) and physical activity are becoming significant outcome parameters. We prospectively assessed these parameters in patients with autoimmune and nonautoimmune liver disorders undergoing LTx. *Materials and Methods.* Patients (*n* = 107) were subdivided into 3 groups depending on the time after LTx: group-A (*n* = 21): 6–12 months; group-B (*n* = 48): 13–36 months; and group-C (*n* = 38): >37 months. SF-36 and IPAQ were applied in HRQoL and physical activity assessment. *Results.* Females had impaired HRQoL in most SF-36 domains. Younger patients showed higher scores at SF-36 *physical functioning* domain but IPAQ was not influenced by age. Group-B had higher *general health* and *physical component summary* than group-A (*P* = 0.037, *P* = 0.04, resp.) and total IPAQ than group-C (*P* = 0.047). The *sitting time* domain was longer in group-A than in group-B and group-C (*P* = 0.0157;  *P* = 0.042, resp.). Employed patients had better HRQoL and higher physical activity than those not working. SF-36 and IPAQ were unrelated to the autoimmune etiology of liver disease. *Conclusions.* These findings show that female and unemployed patients have worse HRQoL, while gender and age at LTx time do not affect IPAQ's physical activity. The autoimmune etiology of liver disease does not influence HRQoL and physical activity after LTx.

## 1. Introduction

The final outcome of autoimmunity and the extent by which it progresses to clinically overt disease have a direct or indirect impact and/or are influenced by parameters associated with health-related quality of life (HRQoL). HRQoL is a multidimensional concept that includes subjective evaluations of domains related to physical, mental, emotional, and social functioning in a context of a disease or disability and their treatment [1]. An evaluation of HRQoL denotes an effort to define how variables within the dimension of health relate to specific measurements that have been found to be of importance to subjects [2]. With the improvement of medical treatments followed by prolongation of survival, HRQoL has emerged as an important clinical issue. Several studies have demonstrated that the HRQoL in patients with chronic liver conditions is significantly impaired [3–6]. This refers in particular to those subjects with liver cirrhosis complicated with hepatic encephalopathy, ascites, or pruritus [7–10]. However, several HRQoL parameters are also impaired in noncirrhotic patients with chronic liver diseases, such as those suffering from primary biliary cirrhosis (PBC) [11–13]. We have recently shown that certain genes related with immune dysregulation are tightly involved in the impairment of HRQoL in PBC, long before the need for LTx [14]. Quality of life improves considerably after liver transplantation (LTx); nevertheless, transplant recipients show lower HRQoL scores than the general population [15]. Regular physical activity has been demonstrated to be of significance in long-term recovery process after LTx and to positively affect quality of life [16]; however, many liver transplant recipients are sedentary [17, 18]. A low physical activity contributes to the development of posttransplant metabolic abnormalities and cardiovascular complications, which are the third leading cause of long-term mortality after LTx [17, 19]. To date, there are limited data on factors related to low physical activity and impaired HRQoL in liver transplant recipients. We ourselves previously noted that specific factors of HRQoL are significantly impaired in patients with autoimmune liver diseases, and in particular in patients with primary biliary cirrhosis (PBC), an autoimmune cholestatic disease [11]. Patients who require LTx due to autoimmune liver disorders also include those with primary sclerosing cholangitis (PSC) and—nowadays to a lesser extent—patients affected with autoimmune hepatitis (AIH). Taking into account that most patients requiring LTx are largely subdivided to those with autoimmune liver diseases, viral hepatitides, or alcoholic liver disease, we have considered that it would be of interest to investigate the factors that affect HRQoL in transplanted patients, paying special attention to those with autoimmune liver diseases. This could give us insight as to whether autoimmunity may have a profound effect in HRQoL and physical activity factors that could be distinguished from those seen in patients with nonautoimmune liver conditions. Our consecutive cohort of LTx patients offered us two unique opportunities: first, to study HRQoL in undivided LTx recipients without paying special attention to the underlying liver disease. This has given us the opportunity to assess prospectively several factors that affect HRQoL and physical functions in patients who underwent LTx in our centre. We also stratified our patients to those with autoimmune and nonautoimmune liver diseases and compared their characteristics.

## 2. Materials and Methods

### 2.1. Patients

One hundred and seven (62 males/45 females) consecutive liver transplant recipients reviewed in our Out-Patient Clinic were included in the study between June 2011 and October 2012. All patients were enrolled at least 6 months after the surgical procedure. Patients were divided into 3 groups, depending on the period after LTx: (group-A, *n* = 21): 6–12 months after LTx; (group-B, *n* = 48): 13–36 months after LTx; and (group-C, *n* = 38): more than 37 months after LTx. Indications for LTx included the following etiologies: alcoholic (*n* = 24), chronic autoimmune cholestatic liver diseases (PBC and PSC, total *n* = 23), AIH (*n* = 10), viral hepatitides (*n* = 15, 11 chronic hepatitis C, 4 chronic hepatitis B), and various other causes of liver disease (*n* = 35).

The demographic data of the participating patients are summarized in Table 1.

The patients under investigation were clinically stable and did not suffer from LTx-related complications and severe comorbidities, which could influence their HRQoL or physical activity, such as malignancy, decompensated diabetes mellitus, renal insufficiency requiring dialysis, heart failure ≥ NYHA II, physical disability, orthopedic disorders, rheumatoid arthritis, or pulmonary disease. The mean body mass index (BMI) of included subjects was 27.0 ± 5.0 kg/m^2^. Thirty-eight (35.5%) patients were overweight (BMI between 25 and 30 kg/m^2^) and 23 (21.5%) were obese (BMI ≥ 30 kg/m^2^).

Forty patients (37.4%) were employed at the time of the survey. Among 67 subjects, who were not working, 21 (31.3%) were retired pensioners and 46 (68.7%) were disability pensioners. Patients with an active profession were younger than patients who did not work (retired or disability pensioners) (45.7 ± 12.8 versus 52.5 ± 9.8 years, *P* = 0.003). The two groups, however, did not differ in relation to gender and the time duration after LTx (data not shown).

### 2.2. Assessment of Health-Related Quality of Life

HRQoL was assessed with the Medical Outcomes Study Short Form (SF-36). The SF-36 is the most widely used and extensively validated generic questionnaire for the assessment of HRQoL in various clinical settings and different populations [20]. It consists of 36 items, grouped in 8 domains, in the area of physical health (domains: *physical functioning*,* role limitations due to physical problems*, *bodily pain,* and *general health*) and mental health (domains: *vitality, social functioning*,* role limitations due to emotional problems*, and *mental health*). Each domain is scored between 0 to 100 points, with higher scores indicating better HRQoL. Two summary scores, *physical component* and *mental component*, are obtained as a mean value calculated based on the corresponding domains. This study was carried out under a licensed approval certificate (CT132326/OP012559) for the use of the SF-36 questionnaire.

### 2.3. Assessment of Physical Activity

For the assessment of physical activity the Polish long version of International Physical Activity Questionnaire (IPAQ) was used [21]. IPAQ is an instrument for monitoring levels of physical activity of an adult population between 15 and 69 years and was developed for surveillance activities and to guide policy development related to health-enhancing physical activity across various life domains. The long version of the IPAQ comprises 27 items and investigates 4 physical activity domains (work, leisure, chores, and transport), as well as time spent sitting as a proxy for sedentary behavior [22]. Physical activity is reported as a continuous score, by domain and by intensity of physical activity (moderate or vigorous), or for *walking*. Physical activity energy expenditure is calculated according to the following formula (in MET-min*·*week-1): number of days spent doing the activity × average duration of the activity per day × energy cost of the activity. The energy cost of an activity is expressed in MET (metabolic equivalent task). A MET is the ratio of the energy expenditure during a given activity divided by the resting energy expenditure [23].

The following MET values were drawn from the scoring protocol: 3.3 for *walking*, 4 for moderate intensity physical activity, 8.0 for vigorous physical activity, 6.0 for cycling, 5.5 for vigorous physical activity in the garden or yard, and 3.0 for domestic activities [24].

### 2.4. Ethics

Written informed consent was obtained from each patient included in the study. The study protocol was approved by the appropriate Ethics Committee of Pomeranian Medical University and conformed to the ethical guidelines of the 1975 Declaration of Helsinki (6th revision, 2008).

### 2.5. Statistics

Data are shown as means and standard deviations. The SF-36 and IPAQ scores were correlated with various clinical factors of potential significance. Data were analyzed using Stat-View-5 Software (SAS Institute, Cary, NC, USA) using Fisher's exact and ANOVA analysis. Categorical data were compared using Levene's test for equality of variances and both pooled-variances and separate-variances *t*-tests for equality of means. Correlations were tested with Pearson Correlation test. *P* value <0.05 was considered statistically significant.

## 3. Results

### 3.1. Quality of Life

Age was comparable between female and male patients at the time of the survey (50.7 ± 11.9 versus 49.4 ± 11.1 years, respectively; *P* = 0.56); still, all but two (*role limitations due to physical problems *and* general health*) domains of SF-36 HRQoL were worse in females compared to males (Table 2(a)). The most profound impairment was noted in the domains of *bodily pain* (*P* = 0.0004) and *role limitations due to physical problems* (*P* = 0.0001).

Younger patients scored higher at *physical functioning* domain (*r* = −0.295, *P* = 0.002). That domain subsequently showed a significantly negative correlation with the age at LTx (*r* = −0.297, *P* = 0.002). Regarding the time period after LTx, a significant improvement in group-B compared to group-A was seen in terms of *general health* (*P* = 0.037) and *physical component summary* (*P* = 0.04) domains (Table 2(b)). The underlying cause of the disease/indication for LTx had no effect on HRQoL (data not shown). Active workers had significantly better HRQoL in most domains of physical aspect of well-being, including *physical functioning, role limitations due to physical problems, bodily pain, physical component summary,* and *role limitations due to emotional problems* compared to pensioners due to retirement or disability (Table 3(a)). BMI correlated negatively with *physical functioning* domain (*r* = −0.221, *P* = 0.022) and *physical component summary* (*r* = −0.207, *P* = 0.032). Obese patients (BMI ≥ 30 kg/m^2^) showed significantly reduced HRQoL in domains: *physical functioning*, *general health,* and *physical component summary* (Table 3(b)).

### 3.2. Physical Activity

Amongst analyzed patients, distribution of activity was as follows: *total activity*: 4497.1 ± 3375.7 MET-min/week (range: 1056.0–22740.0 MET-min/week), *walking*: 1286.4 ± 910.8 MET-min/week (range: 0.0–4158.0 MET-min/week), *moderate activity*: 2402.9 ± 1368.7 MET-min/week (range: 180.0–6930.0 MET-min/week), *vigorous activity*: 807.9 ± 3151.7 MET-min/week (range: 0.0–21480.0 MET-min/week), and *sitting time  *633.5 ± 181.3 min-week (range: 300.0–1080.0 min-week). Eighty-four patients (44 males) did not undertake any *vigorous activity*. No difference in physical activity between males and females both in terms of total and specific domains of IPAQ was seen, except of *vigorous activity* (Table 4(a)). No correlation was observed with IPAQ and age at survey or age at surgery and the underlying cause of the disease/indication for LTx (data not shown). Group-B was more active with regard to total IPAQ (5333.4 ±4553.7 MET-min/week) versus group-C (3882.6 ± 1538.9 MET-min/week, *P* = 0.047). Group-B tended to be more active than group-A (3697.6 ± 2053.3 MET-min/week, *P* = 0.06, Table 4(b)). As expected, *sitting time* domain was significantly longer in group-A (720.0 ± 171.8 min-week) than in group-B (605.8 ± 174.5 min-week, *P* = 0.0157) and group-C (620.5 ± 184.7 min-week, *P* = 0.042, Table 4(b)). Participants who commenced an employment after LTx showed significantly higher physical activity in total IPAQ and in *vigorous activity* domains in comparison to patients out of work (*P* = 0.005 and *P* = 0.0014, resp., Table 5(a)). There was a trend towards a negative correlation between lower *total activity* and BMI (*r* = −0.174, *P* = 0.07). In comparison to patients with ideal weight, overweight patients were less active in relation to *total activity* and *vigorous activity* (*P* = 0.0082 and *P* = 0.047, resp.), and obese subjects showed reduced *total activity* (*P* = 0.027, Table 5(b)).

### 3.3. Relationship between Physical Activity and Quality of Life after Liver Transplantation

From all domains of SF-36 questionnaire, only *social functioning* domain correlated negatively with *sitting time* domain of IPAQ (*r* = −0.203, *P* = 0.036).

### 3.4. HRQoL and Physical Function Test Parameters in Patients with Autoimmune and Nonautoimmune Liver Diseases

As we mentioned, the indication for LTx did not affect HRQoL and physical activity. We then compared patients with underlying autoimmune liver diseases (AIH, PBC, and PSC) with those without autoimmune liver diseases, in relation to specific SF-36 HRQoL and IPAQ physical activity performance. When LTx patients were stratified according to the presence of autoimmune liver disease or not as the cause of the underlying disease, we did not observe any differences in terms of well-being or physical activity (Table 6).

## 4. Discussion

Several studies have shown that HRQoL improves considerably after LTx. Nevertheless, transplant recipients demonstrate lower HRQoL scores and remain less physically active than the general population [25–28]. In this study, we prospectively assessed the factors that can influence daily living and physical behaviors in 107 patients, who underwent LTx in our centre.

In subgroup analysis, we found that the cause of transplantation did not influence physical activities and perception of daily life (Table 5). This is an important topic because a substantial group of patients with liver diseases (in our cohort 31%) stems from an underlying autoimmune disease (PBC, PSC, or AIH), and this could help us delineate the extent by which the autoimmune nature of the original disease may impact the overall performance scores. Our analysis suggests that the mechanisms leading to end-stage liver failure and LTx are irrelevant to the HRQoL and physical activity of patients who have undergone LTx. On the other hand, older age was a factor significantly impairing HRQoL, but this feature is also seen in the general population and by no means can be seen as a major contributing factor [29]. As was expected, we observed that patients in their first 6–12 months after LTx demonstrate more sedentary habits; such features are most likely related to recovery and fear of pain due to physical efforts. In contrast, patients assessed in their second and third year after LTx showed increasing physical activity and improved physical aspects of well-being such as *general health* and *physical component summary* of SF-36. An apparent explanation for this finding is that full recovery enables these patients to perform better in terms of more demanding physical effort and active lifestyle needs [17]. Gross et al. have reported similar findings and noted that limitations in several activities due to patients' role and physical health problems virtually cease a year after LTx [27]. Somewhat surprisingly, this trend was not observed in LTx patients studied 36 months or after the procedure. In these patients, there was no further improvement in the quality of their life, while physical activity was again significantly decreased. The long-term impairment of physical functions in liver transplant recipients has also been noted by other investigators [15]. While the overall perception of health improves after LTx, a significant proportion of patients suffer from chronic complaints such as chronic pain or fatigue, which restricts their physical activity [30–33]. However, in our group of patients we did not observe such unwanted symptoms (low scores at *bodily pain* SF-36 domain).

Data assessing the impact of physical activity on nutritional status in liver transplant recipients are limited. Kallwitz et al. showed that the metabolic syndrome is common after LTx with higher prevalence in patients over 1 year after LTx, and is inversely correlated with exercise intensity [18]. Published data showed an association of higher activity levels with lower rates of hypertension and a lower BMI [16]. We found an obese or overweight habitus strongly associated with reduced physical activity and impaired physical components of HRQoL, but the interpretation of our findings is not an easy task as we do not know whether physical inactivity (and lower HRQoL scores) precedes or follows that of increased BMI.

Patients doing their professional work had significantly better scores of SF-36, mainly related to physical aspects of well-being (i.e., *physical functioning, role limitations due to physical problems, bodily pain, physical component summary,* and* role limitations due to emotional problems*) and were significantly more physically active than those out of work (pensioners). These observations are in agreement with previous studies, in which persons that had an active work life had significantly better HRQoL compared to those out of work [15, 34–36]. Liver transplant recipients more frequently experience fatigue and demonstrate lower SF36 *physical functioning* scores than those engaged in any educational or working activity [37]. This has been attributed to the fact that professional work provides external stimuli and allows the return to normal lifestyle and social integration [38]. Also, fulfillment of professional tasks and work satisfaction enhances the sense of independence. Thus, patients who remain out of work may potentially be less motivated and have more impaired physical activity than those with an active work life.

Perhaps the most striking finding of this analysis is a significantly worse HRQoL in female patients in the majority of SF-36 domains. This new finding contrasts published data reporting comparable HRQoL scores between males and females. In agreement with our data, Cowling et al. found better quality of life in male subjects using their own HRQoL questionnaire, specifically designated for LTx patients [39]. In other disciplines, HRQoL is worse in female patients with coronary artery disease than in men [40], while women with early rheumatoid arthritis perform better in terms of HRQoL than their male counterparts [41]. Gender-related differences on quality of life parameters are complex and deserve thorough investigation.

We are aware of the constraints of the present study. For example, recurrence of the original disease including viral hepatitides, PBC, or PSC may affect HRQoL parameters. Amongst the 11 chronic hepatitis C patients, 5 had recurrent disease which required initiation of antiviral treatment including two who achieved sustained virological response but the treatment finished 4 years and 1 year before the inclusion in the study, respectively. Thus, it is rather unlikely that recurrence is a precipitating factor which could affect the current analyses, though such an effect cannot be excluded. Another limitation is that the study did not include data on healthy subjects and is cross-sectional in nature and not longitudinal study. Nevertheless, it provides important information regarding the impact of environment in the form of HRQoL and physical activity features after LTx. Our findings suggest that the indication for transplantation (autoimmune or nonautoimmune) does not influence the quality of life but emphasizes the role of employment in complete recovery and return to normal life. We underline differences amongst gender in perception of daily life and a profound impairment of SF-36 scores in females.

## Figures and Tables

**Table 1:** Demographic and clinical data on analyzed patients.

Total number of patients	*n* = 107
Age at transplantation [years, mean ± SD (range)]	46.7 ± 11.6 (17–63)
Age at survey [years, mean ± SD (range)]	49.9 ± 11.4 (21–67)
Gender [male/female]	62 (57.9%)/45 (42.1%)
BMI [kg/m^2^, mean ± SD (range)]	27.0 ± 5.0 (18.0–43.0)
Period after transplantation	
Group-A (6–12 months)	21 (19.6%)
Group-B (13–36 months)	48 (44.9%)
Group-C ( >36 months)	38 (35.5%)
Employment (yes/no)	40 (37.4%)/67 (62.6%)
Original diagnosis	
Alcohol liver disease	24 (22.4%)
Viral hepatitides	15 (14.0%)
Autoimmune hepatitis	10 (9.4%)
Autoimmune cholestatic (PBC, PSC)	23 (21.5%)
Other*	35 (32.7%)

Group-A: 6 months to 12 months after liver transplantation; group-B: from 12 months to 36 months after liver transplantation; group-C: over 36 months after liver transplantation; *other reasons for LTx included cryptogenic cirrhosis (*n* = 8); Fulminant Wilson's (*n* = 5), chronic Wilson's (*n* = 2); non-A, non-B hepatitis (*n* = 7); amanita poisoning (*n* = 7); Budd-Chiari syndrome (*n* = 4); secondary biliary cirrhosis (*n* = 1) and acute fatty liver of pregnancy (*n* = 1). BMI: body mass index; PBC: primary biliary cirrhosis; PSC: primary sclerosing cholangitis.

**Table:** (a)

SF 36	Males (*n* = 62)	Females (*n* = 45)	*P* value
PF	78.5 ± 22.2	63.9 ± 22.5	0.001
RP	67.6 ± 26.2	58.6 ± 20.0	0.05
BP	79.4 ± 25.4	60.5 ± 28.2	0.0004
GH	61.9 ± 20.2	56.3 ± 18.4	0.14
VT	64.9 ± 16.1	55.8 ± 18.4	0.0077
SF	79.4 ± 20.4	66.1 ± 79.4	0.0023
RE	81.5 ± 20.0	65.2 ± 21.5	0.0001
MH	71.1 ± 15.4	62.1 ± 17.4	0.0057
PCS	48.2 ± 9.1	43.7 ± 7.6	0.0073
MCS	48.9 ± 8.2	43.1 ± 9.9	0.0015

**Table:** (b)

SF 36	A (*n* = 21)	B (*n* = 48)	C (*n* = 38)	*P* (A versus B)	*P* (A versus C)	*P* (B versus C)
PF	66.7 ± 25.1	71.8 ± 25.7	76.2 ± 18.7	0.405	0.136	0.386
RP	55.4 ± 24.9	65.6 ± 23.0	66.3 ± 24.8	0.105	0.096	0.899
BP	62.2 ± 25.2	74.2 ± 27.7	73.0 ± 29.6	0.105	0.159	0.845
GH	52.7 ± 18.5	63.4 ± 18.5	58.4 ± 20.7	**0.037**	0.280	0.239
VT	61.0 ± 15.3	60.6 ± 17.9	61.8 ± 18.8	0.920	0.864	0.737
SF	75.6 ± 21.1	77.1 ± 23.7	68.8 ± 21.7	0.801	0.266	0.091
RE	68.7 ± 25.4	75.2 ± 22.2	77.2 ± 19.8	0.261	0.157	0.674
MH	67.9 ± 11.7	67.0 ± 17.3	67.5 ± 18.8	0.839	0.938	0.882
PCS	42.5 ± 8.8	47.3 ± 8.4	47.2 ± 8.9	**0.04**	0.051	0.967
MCS	47.0 ± 8.6	46.6 ± 9.6	46.9 ± 9.8	0.8819	0.6964	0.7569

All values are shown as mean ± SD.

Group-A: 6 months to 12 months after liver transplantation; group-B: from 12 months to 36 months after liver transplantation; group-C: over 36 months after liver transplantation.

PF: physical functioning; RP: role limitations due to physical problems; BP: bodily pain; GH: general health; VT: vitality; SF: social functioning; RE: role limitations due to emotional problems; MH: mental health; PF: physical functioning; PCS: physical component score; MCS: mental component score.

**Table:** (a)

SF 36	Employed (*n* = 40)	Pensioners (*n* = 67)	*P* value
PF	**81.3 ± 19.6**	**67.0 ± 23.9**	**0.002**
RP	**72.3 ± 23.3**	**58.8 ± 23.4**	**0.004**
BP	**81.1 ± 24.4**	**65.7 ± 28.7**	**0.005**
GH	61.6 ± 23.4	58.3 ± 16.9	0.407
VT	63.5 ± 17.1	59.7 ± 17.8	0.298
SF	77.8 ± 22.4	71.5 ± 22.6	0.161
RE	**83.5 ± 18.2**	**69.3 ± 22.6**	**0.001**
MH	71.0 ± 17.3	65.2 ± 16.2	0.083
PCS	**49.4 ± 8.6**	**44.5 ± 8.4**	**0.005**
MCS	48.3 ± 8.6	45.4 ± 9.7	0.113

**Table:** (b)

SF 36	1 (*n* = 46)	2 (*n* = 38)	3 (*n* = 23)	*P* (1 versus 2)	*P* (1 versus 3)	*P* (2 versus 3)
PF	**74.7 ± 23.5**	**77.4 ± 19.8**	**59.3 ± 24.7**	0.59	**0.0089**	**0.0031**
RP	65.1 ± 24.5	66.8 ± 24.1	56.5 ± 22.96	0.75	0.17	0.11
BP	71.9 ± 27.9	74.3 ± 29.03	65.6 ± 26.9	0.69	0.38	0.25
GH	60.4 ± 19.7	**63.5 ± 21.1**	**51.3 ± 14.1**	0.46	0.07	**0.017**
VT	60.9 ± 20.2	63.5 ± 15.5	57.6 ± 15.2	0.49	0.47	0.21
SF	71.2 ± 24.3	75.98 ± 21.8	75.5 ± 20.8	0.34	0.45	0.94
RE	73.4 ± 24.6	76.1 ± 19.8	74.6 ± 21.1	0.58	0.82	0.80
MH	66.2 ± 16.7	70.2 ± 18.1	64.8 ± 14.7	0.28	0.74	0.22
PCS	**47.3 ± 8.1**	**48.01 ± 9.3**	**41.5 ± 7.8**	0.71	**0.0081**	**0.0043**
MCS	45.2 ± 10.6	47.3 ± 8.6	47.5 ± 8.04	0.32	0.36	0.95

All values are shown as mean ± SD.

Group 1: normal BMI (18–25 kg/m^2^), group 2: overweight (BMI 25–30 kg/m^2^), group 3: obese (BMI 25–30 kg/m^2^).

PF: physical functioning; RP: role limitations due to physical problems; BP: bodily pain; GH: general health; VT: vitality; SF: social functioning; RE: role limitations due to emotional problems; MH: mental health; PF: physical functioning; PCS: physical component score; MCS: mental component score.

**Table:** (a)

IPAQ	Males (*n* = 62)	Females (*n* = 45)	*P*
Total (MET-min/week)	4912.8 ± 4027.8	3924.4 ± 2093.3	0.136
Walking (MET-min/week)	1222.9 ± 794.8	1373.9 ± 1053.3	0.399
Moderate (MET-min/week)	2342.2 ± 1322.9	2486.5 ± 1440.2	0.593
Vigorous (MET-min/week)	**1347.7 ± 4064.9**	**64.0 ± 212.1**	**0.03**
Sitting time (min-week)	621.9 ± 176.1	649.3 ± 189.0	0.443

**Table:** (b)

IPAQ	A (n = 21)	B (*n* = 48)	C (*n* = 38)	*P* (A versus B)	*P* (A versus C)	*P* (B versus C)
Total (MET-min/week)	3697.6 ± 2053.3	5333.4 ± 4553.7	3882.6 ± 1538.9	0.06	0.83	**0.047**
Walking (MET-min/week)	1216.2 ± 890.5	1350.6 ± 1027.7	1244 ± 772.4	0.577	0.912	0.594
Moderate (MET-min/week)	2119.5 ± 1158.7	2548.7 ± 1601.4	2375.4 ± 1144.3	0.235	0.494	0.562
Vigorous (MET-min/week)	361.9 ± 1568.0	1464.2 ± 4506.9	263.2 ± 632.6	0.193	0.91	0.08
Sitting time (min-week)	720.0 ± 171.8	605.8 ± 174.5	620.5 ± 184.7	**0.016**	**0.042**	0.7

All values are shown as mean ± SD.

Group-A: 6 months to 12 months after liver transplantation; group-B: from 12 months to 36 months after liver transplantation; group-C: over 36 months after liver transplantation.

**Table:** (a)

IPAQ	Employed (*n* = 40)	Pensioners (*n* = 67)	*P*
Total (MET-min/week)	5668.2 ± 4787.0	3797.9 ± 1852.8	**0.005**
Walking (MET-min/week)	1287.5 ± 870.9	1287.5 ± 985.5	0.98
Moderate (MET-min/week)	2340.7 ± 1451.7	2440.0 ± 1326.5	0.71
Vigorous (MET-min/week)	2043.0 ± 4934.6	70.5 ± 296.9	**0.0014**
Sitting time (min-week)	632.5 ± 173.2	634.0 ± 187.2	0.96

**Table:** (b)

IPAQ	1 (*n* = 46)	2 (*n* = 38)	3 (*n* = 23)	*P* (1 versus 2)	*P* (1 versus 3)	*P* (2 versus 3)
Total (MET-min/week)	**5585.3 ± 4599.4**	**3651.9 ± 1635.4**	**3717.3 ± 1670.0**	**0.0082**	**0.027**	0.93
Walking (MET-min/week)	1361.2 ± 1179.2	1283.1 ± 686.8	1142.1 ± 571.1	0.70	0.35	0.56
Moderate (MET-min/week)	2600.5 ± 1294.0	2109.8 ± 1337.5	2491.7 ± 1534.2	0.10	0.75	0.29
Vigorous (MET-min/week)	**1623.5 ± 4674.7**	**258.9 ± 567.3**	83.5 ± 400.3	**0.047**	0.054	0.83
Sitting time (min-week)	663.5 ± 182.9	593.7 ± 183.9	639.1 ± 168.4	0.08	0.59	0.34

All values are shown as mean ± SD.

Group 1: normal BMI (18–25 kg/m^2^); group 2: overweight (BMI 25–30 kg/m^2^); group 3: obese (BMI 25–30 kg/m^2^).

**Table 6:** Quality of life and physical activity after liver transplantation in patients with autoimmune versus nonautoimmune etiology of liver disease.

	Autoimmune (*n* = 33)	Nonautoimmune (*n* = 74)	*P* value
SF 36			
PF	69.1 ± 23.4	73.8 ± 23.3	0.339
RP	62.1 ± 23.3	64.6 ± 24.6	0.624
BP	66.5 ± 27.7	73.6 ± 28.1	0.232
GH	56.8 ± 21.4	60.8 ± 18.7	0.335
VT	60.4 ± 16.9	61.4 ± 18.0	0.790
SF	72.0 ± 19.0	74.7 ± 24.1	0.572
RE	72.2 ± 21.8	75.7 ± 22.2	0.457
MH	66.2 ± 17.8	67.8 ± 16.4	0.650
PCS	44.9 ± 8.9	47.0 ± 8.7	0.256
MCS	46.0 ± 8.6	47.7 ± 9.8	0.732

IPAQ			
Total (MET-min/week)	4464.4 ± 3370.3	4511.7 ± 3401.0	0.947
Walking (MET-min/week)	1483.0 ± 901.6	1198.7 ± 907.2	0.137
Moderate (MET-min/week)	2077.1 ± 1043.8	2548.2 ± 1474.1	0.100
Vigorous (MET-min/week)	904.2 ± 3147.2	764.9 ± 3174.2	0.834
Sitting time (min/week)	620.0 ± 182.9	639.5 ± 181.5	0.610

All values are shown as mean ± SD.

PF: physical functioning; RP: role limitations due to physical problems; BP: bodily pain; GH: general health; VT: vitality; SF: social functioning; RE: role limitations due to emotional problems; MH: mental health; PF: physical functioning; PCS: physical component score; MCS: mental component score.
